# The Time-Domain Integration Method of Digital Subtraction Angiography Images

**DOI:** 10.1155/2018/5284969

**Published:** 2018-09-30

**Authors:** Shuo Huang, Le Cheng, Bin Zhu, Ping Zhou, Yu Sun, Bing Zhang, Suiren Wan

**Affiliations:** ^1^School of Biological Sciences and Medical Engineering, Southeast University, Nanjing 210096, China; ^2^Shanghai United Imaging Healthcare Co., Ltd., Shanghai 201807, China; ^3^Department of Radiology, The Affiliated Drum Tower Hospital of Nanjing University Medical School, Nanjing 210008, China; ^4^Institute of Cancer and Genomic Science, University of Birmingham, Birmingham B15 2TT, UK

## Abstract

The clarity improvement and the noise suppression of digital subtraction angiography (DSA) images are very important. However, the common methods are very complicated. An image time-domain integration method is proposed in this study, which is based on the blood flow periodicity. In this method, the images of the first cardiac cycle after the injection of the contrast agent are integrated to obtain the time-domain integration image. This method can be used independently or as a postprocessing method of the denoising method on the signal image. The experimental results on DSA data from an aortic dissection patient show that the image time-domain integration method is efficient in image denoising and enhancement, which also has a good real-time performance. This method can also be used to improve the denoising and image enhancement effect of some common models.

## 1. Introduction

Circulatory system diseases, such as aortic dissection, have been the focus of medical research [1, 2] for their dangerousness and high incidence. Improving the clarity of the captured medical image helps us to diagnose more accurately, which is an important research field.

Digital subtraction angiography (DSA), as a real-time approach, is commonly employed in the clinical diagnosis of circulatory system disease [3, 4], especially in the real-time surgical monitoring and the medical examination among small branches of blood vessels which are difficult to be measured by other methods. In order to protect the patient, it is important to shorten the shooting time and to reduce the dosage of contrast media when capturing the images.

A lot of image denoising methods have been proposed in recent years, but they are all problematic when applied to the DSA images. For example, the image reconstruction method based on the level set theory [5], wavelet decomposition and reconstruction method [6, 7], Bayesian method [8], and image denoising method based on anisotropic diffusion [9] generally need a long operation time, which cannot meet the real-time requirements of DSA image processing. Moreover, images processed by these approaches are usually not clear enough to show the details such as edges and textures. In 2004, Candes et al. [10] proposed an image denoising method based on the sparse decomposition. On this basis, Needell and Vershynin [11] proposed the regularized orthogonal matching pursuit (ROMP) method; Scholefield and Dragotti [12] used a sparse quadtree decomposition representation to remove the noise in images; Adler et al. employed the shrinkage learning approach to acquire the high-resolution reconstruction image [13–17]. However, the operation of these approaches is also very complicated. Therefore, it is necessary to find an image processing method which is more suitable for the real-time analysis of DSA images.

In this work, an image time-domain integration method based on blood flow periodicity has been proposed. In this algorithm, the DSA images of the first cardiac cycle after the injection of the contrast agent are extracted denoised by the wavelet reconstruction method firstly, and then these images are integrated to obtain the time-domain integration image, which is named after the TDI image in this paper. This method contributes to the diagnosis of circulatory system diseases.

## 2. Materials and Methods

### 2.1. The Noise Model

The theoretical gray-scale *wj*(*x*_0_, *y*_0_) at a certain pixel (*x*_0_, *y*_0_) on the *j*-th frame of DSA images can be obtained from the following equation by the Lambert–Beer law [18, 19]:

(1a) $$\begin{array}{c} w_{j}\left(x_{0},y_{0}\right)=-\ln\left(\frac{I_{j}\left(x_{0},y_{0}\right)}{I_{0}\left(x_{0},y_{0}\right)}\right)=-\ln\left(e^{-k\times {∭}_{V_{j}\left(x_{0},y_{0}\right)}c\left(V_{j}\right)dV}\right)=k\times N_{j}\left(x_{0},y_{0}\right), \end{array}$$

where *I*_0_ and *I*_r_ are the X-ray transmission amount before and after the addition of the contrast agent, respectively. *V*_*j*_(*x*_0_, *y*_0_) is the volume of blood vessels at pixel (*x*_0_, *y*_0_). *Nj*(*x*_0_, *y*_0_) and *c*(*V*_*j*_) are the number and amount of substance concentration of contrast agent particles at pixel (*x*_0_, *y*_0_), respectively. *k* donates the absorption coefficient.

The image quality degrades in the original DSA image, as a result of the limitations on the imaging system's resolution and the influence of additive noise such as Gaussian noise, which is donated by **w**_**j**_ and can be expressed in as follows [6]:

(1b) $$\begin{array}{c} W_{j}=w_{j}\;*\;h_{j}+n_{j}, \end{array}$$

where **h**_**j**_ and **n**_**j**_ represent the point spread function and the additive noise, respectively. Operator “*∗*” is the convolution operator. Equation (1b) can be rewritten to matrix form using the block Toeplitz matrix **H**_**j**_, as shown in the following equation:

(1c) $$\begin{array}{c} W_{j}=w_{j}\cdot H_{j}+n_{j}. \end{array}$$

It is difficult to solve Equation (1c) when only **W**_**j**_ is given. However, since the gray-scale level of a certain pixel is proportional to the number of contrast agent particles in that pixel, the number of contrast agent particles follows the motion pattern of blood. And as for the blood motion pattern, on consideration of the periodicity of human heartbeat, the blood flow rate in human body is also cyclical, which can be expressed in the following equation:

(2) $$\begin{array}{c} v_{b}\left(nT+t\right)=\bar{v_{b}}\left(t\right)+\Psi \left(\bar{v_{b}}\right),\;t\in Z, \end{array}$$

where **v**_**b**_(*nT*+*t*) is the velocity field of blood at time (*nT*+*t*). $\bar{v_{b}}\left(t\right)$ denotes the average velocity field of blood at time *t*, which is the mean flow velocity at the same time in multiple cardiac cycles. *T* is the length of the cardiac cycle. $\Psi \left(\bar{v_{b}}\right)$ characterizes the changes in flow velocity owing to factors such as the instability of human blood pressure. $\Psi \left(\bar{v_{b}}\right)$ can be regarded as a zero-mean-value distribution with a small variance, since patients are under the total anesthesia during the shooting process and their vital signs remain stable. According to the Wilke–Chang equation [20], the free diffusion rate of the contrast agent in the blood is much smaller than the blood flow rate, and thus the contrast agent obeys the same movement law as the blood. Therefore, the periodicity of the blood flow rate can be employed to improve the clarity of DSA images.

### 2.2. Image Integration

In order to decrease the shoot time and the contrast agent's injection quantity, images in the first cardiac cycle (donated by the cardiac cycle *S*) after the injection of the contrast agent are analyzed in this study. Firstly, the time at which the cardiac cycle begins is set to be *t*=0. Subsequently, the velocity of the *i-*th contrast agent particle in this cardiac cycle is expressed as **u**_**i**_(*t*)=(**u**_**i**_(*t*) · *x*, **u**_**i**_(*t*) · *y*, **u**_**i**_(*t*) · *z*). Since the motion of the contrast agent particles is consistent with that of the blood, and on consideration of the velocity stability shown in Equation (2), the velocity of the particle at each position on its trajectory can be regarded as a sample of the blood flow field at that location. Therefore, once the substantial number of particles is extracted, the average velocity field of the contrast agent at the pixel (*x*_0_, *y*_0_) in the entire cardiac cycle can be estimated by the mean velocity field value of particles which flowed through that pixel during the calculated cardiac cycle, as shown in the following equation:

(3a) $$\begin{array}{c} \bar{v_{c}}\left(x_{0},y_{0}\right)=\frac{1}{N\left(x_{0},y_{0}\right)}\overset{N\left(x_{0},y_{0}\right)}{\underset{i=1}{\sum }}u_{i}\left(t_{i}\right), \end{array}$$

where *t*_*i*_ represents the time when the *i*-th particle approached pixel (*x*_0_, *y*_0_).

The total time length that the *i*-th particle appears in pixel (*x*_0_, *y*_0_) during one cardiac cycle *T* satisfies Equation (3b), where *L*_*i*_(*x*_0_, *y*_0_) represents the distance of the *i*-th particle in the range of pixel (*x*_0_, *y*_0_) and *u*_*i*,//_(*x*_0_, *y*_0_) is set to be the magnitude of velocity component which is parallel to the image plane in pixel (*x*_0_, *y*_0_) during that cardiac cycle since the photographing each DSA image can be regarded as a sample of each particle's location:

(3b) $$\begin{array}{c} T_{i}\left(x_{0},y_{0}\right)=\min\left\{\frac{L_{i}\left(x_{0},y_{0}\right)}{u_{i,//}\left(x_{0},y_{0}\right)},T\right\}. \end{array}$$

A variate *λ* is set to represent the absorption capacity of light in the unit time of a single contrast agent particle. After that, the time-weighted gray-scale value of the *i-*th particle at pixel (*x*_0_, *y*_0_), *p*_*i*_(*x*_0_, *y*_0_), can be expressed by the following equation:

(3c) $$\begin{array}{c} p_{i}\left(x_{0},y_{0}\right)=\lambda \cdot T_{i}\left(x_{0},y_{0}\right). \end{array}$$

On combination of Equations (3a)–(3c), $\bar{v_{b,//}}\left(x_{0},y_{0}\right)$ can be characterized by the sum of the time integral intensities of particles which have appeared in pixel (*x*_0_, *y*_0_) during the cardiac cycle, as shown in the following equation:

(3d) $$\begin{array}{c} \overset{N\left(x_{0},y_{0}\right)}{\underset{i=1}{\sum }}p_{i}\left(x_{0},y_{0}\right)=\lambda \cdot \overset{N\left(x_{0},y_{0}\right)}{\underset{i=1}{\sum }}T_{i}\left(x_{0},y_{0}\right)=\lambda \cdot N\left(x_{0},y_{0}\right)\cdot \frac{\bar{L}\left(x_{0},y_{0}\right)}{\bar{v_{b,//}}\left(x_{0},y_{0}\right)}, \end{array}$$

where $\bar{L}$ is the average moving distance of the contrast agent particles within that pixel. Since the size of a pixel is small, $\bar{L}$ is approximately equal to the length of each pixel, *L*_pixel_. According to Equations (1a) and (3d), when the frame rate *M* tends to infinity, the following equation can be obtained:

(3e) $$\begin{array}{c} \overset{N\left(x_{0},y_{0}\right)}{\underset{i=1}{\sum }}p_{i}\left(x_{0},y_{0}\right)=\overset{N\left(x_{0},y_{0}\right)}{\underset{i=1}{\sum }}\left(\lambda \times T_{i}\left(x_{0},y_{0}\right)\right)=k\times \overset{M-1}{\underset{j=0}{\sum }}\left(N_{j}\left(x_{0},y_{0}\right)\times \Delta t\right)=\overset{M-1}{\underset{j=0}{\sum }}\left(w_{j}\left(x_{0},y_{0}\right)\times \Delta t\right)\equiv b\left(x_{0},y_{0}\right),\text{ when }M⟶+\infty \text{ and }\Delta t=\frac{T}{M}⟶0. \end{array}$$

Equation (3e) demonstrates that the overall time-domain integration value of pixel (*x*_0_, *y*_0_), *b*(*x*_0_, *y*_0_), can be expressed as the integral of each picture's gray value at that position in the entire cardiac cycle. On consideration that the time step Δ*t* is short in the actual case, Equation (3e) can be employed in the calculation of the captured DSA images. Therefore, the relationship shown in Equation (3f) can be established. And the image **b** is named after the time-domain integration image or the TDI image:

(3f) $$\begin{array}{c} b\left(x_{0},y_{0}\right)=\overset{M-1}{\underset{j=0}{\sum }}\left(w_{j}\left(x_{0},y_{0}\right)\times \Delta t\right)=\lambda \times L_{\text{pixel}}\times \frac{N\left(x_{0},y_{0}\right)}{\bar{v_{c,//}}\left(x_{0},y_{0}\right)}=\overset{M-1}{\underset{j=0}{\sum }}\left(W_{j}\left(x_{0},y_{0}\right)\times \Delta t\right). \end{array}$$

Furthermore, to strength the denoising effect, the images are denoised by the median filter before the time-domain integration since Ling's work [21] shows that the noiseless image is usually insensitive to a median filter.  Table 1 shows the specific steps of our method.

## 3. Results

**Figure 1:**
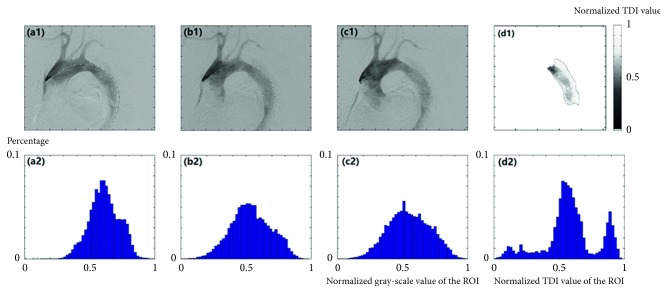
A set of DSA images from a patient.

Figure 1 shows a group of DSA images from a patient with aortic dissection. Figures 1–1 are the DSA images at *t*=1/6*T*, 1/2*T*, and 5/6*T*, respectively. Figure 1 is the TDI image of the dissecting aneurysm extracted by our algorithm. For the sake of comparison, the image of dissecting aneurysm region in Figures 1–1 are extracted and then the normalized gray-scale histograms of dissecting aneurysm regions among Figures 1–1 are obtained, which are shown in Figures 1–1, respectively.  Table 2 shows the mean value, standard deviation, and coefficient of variation of DSA images of the dissecting aneurysm region in one cardiac cycle of the patient in Figure 1.

Table 2 shows that the coefficient of variation of the TDI image is higher than all the DSA images in that cardiac cycle, which means that our TDI image can enhance the details. Moreover, the gap between the peaks in Figure 1 is clearer than those in Figures 1–1, which means that our TDI image has higher resolution than the original images.

## 4. Discussion

Since the blood flow velocity has a certain degree of uncertainty in the actual situation, which directly influenced the gray-scale value of the shot DSA image, Equation (1c) can be rewritten as follows:

(4) $$\begin{array}{c} W_{j}=w_{j}\cdot H_{j}+n_{j}=\left(\left(q_{j}+\Phi_{j}\right)\cdot H_{j}\right)+n_{j}, \end{array}$$

where **q**_**j**_ donates the gray-scale value after the removal of motion randomness.

Equations (3f) and (4) illustrate that the time-domain integration image **b** can be obtained by the following equation:

(5a) $$\begin{array}{c} b=\Delta t\times \left(\overset{M-1}{\underset{j=0}{\sum }}W_{j}\right)=\Delta t\times \left(\overset{M-1}{\underset{j=0}{\sum }}\left(q_{j}+\Phi_{j}\right)\cdot H_{j}+\overset{M-1}{\underset{j=0}{\sum }}n_{j}\right)=\Delta t\times \left(\overset{M-1}{\underset{j=0}{\sum }}\left(q_{j}+G_{j}\right)\right), \end{array}$$

where matrix **G**_**j**_ is defined to characterize the differences between **W**_**j**_ and **q**_**j**_. A new symbol **b**_**x**_ is defined here, which represents the noise-free time-domain integration image. **b**_**x**_=∑_*j*=0_^*M*−1^**q**_**j**_. Then, the error analysis of Equation (5a) is implemented below.

(5b) $$\begin{array}{c} v_{\text{inlet}}\left(t\right)=\left\{\begin{array}{cc} 2.4e^{-7.557t}\sin\left(13.09t\right)\text{,} & 0\leq t\leq 0.24,\\ -0.343e^{-7.557\left(t-0.24\right)}\times \sin\left(13.09\left(t-0.24\right)\right)\left(\text{m/s}\right)\text{,} & 0.24<t<0.2743\left(\text{s}\right)\text{,}\\ 0\text{,} & 0.2743<t\leq 0.8, \end{array}\right. \end{array}$$

(5c) $$\begin{array}{c} \frac{1}{M}\times \overset{M-1}{\underset{j=0}{\sum }}{\left(\frac{G_{j}\left(x_{0},y_{0}\right)}{q_{j}\left(x_{0},y_{0}\right)}\right)}^{2}\geq {\left(\frac{\Delta t\times \sum_{j=0}^{M-1}G_{j}\left(x_{0},y_{0}\right)}{\Delta t\times \sum_{j=0}^{M-1}q_{j}\left(x_{0},y_{0}\right)}\right)}^{2}={\left(\frac{b\left(x_{0},y_{0}\right)-b_{x}\left(x_{0},y_{0}\right)}{b_{x}\left(x_{0},y_{0}\right)}\right)}^{2}, \end{array}$$

(5d) $$\begin{array}{c} {\left\|\underset{M⟶+\infty }{\lim}\left(k\times \overset{M-1}{\underset{j=0}{\sum }}\left(N_{j}\times \frac{T}{M}\right)\right)-b\right\|}_{2}<{\left\|\underset{M⟶+\infty }{\lim}\left(k\times \overset{M-1}{\underset{j=0}{\sum }}\left(N_{j}\times \frac{T}{M}\right)\right)-W_{j}\times T\right\|}_{2}. \end{array}$$

According to Reference [22], when the cardiac cycle length is 0.8 seconds, the blood flow rate at the aortic inlet, denoted by *v*_inlet_, follows Equation (5b). The first part of Equation (5b) denotes the ejection phase, which is followed by a brief closure of blood after the closure of the aortic valve. The flow rate in the rest time is 0. Since the time varies when each particle enters the view field, the time when they arrive at the same position on the image also varies. Therefore, the number of images which satisfy **N**_**j**_(*x*, *y*) ≠ 0 is greater than one in most positions. Thus, Equation (5c) can be obtained.

According to Equation (5c), the signal-to-noise ratio of image **b** (**b**_**x**_ is defined as the noise-free image in this calculation) is higher than the mean signal-to-noise ratio of original DSA images at each pixel. Therefore, the result of our method contains lower noise in the entire view field, and our method can suppress the noise.

Furthermore, Equation (5d) can be deduced from Equation (3e), where ‖‖_2_ stands for the two-norm of matrix. According to Equation (5d), image **b** is closer to the noise-free TDI image **b**_**x**_ than all of the DSA images, which means that image **b** has the highest clarity. Thus, the image **b** can improve the clarity of the original DSA images.

## 5. Conclusion

In summary, this study presents a DSA image denoising and enhancement method based on the periodicity of blood flow. Firstly, the DSA images are reconstructed through the median filter, and then DSA images in a cardiac cycle are integrated and the overall time-domain integration image **b** is obtained. According to the mathematical derivation as well as the verification using aortic dissecting aneurysm images, this study demonstrates that the TDI image of the contrast agent has a lower overall noise than original DSA images, and it is also clearer than the original image. This method can contribute to the feature location extraction and disease prophylaxis in circulatory disease, such as the first break's position extraction of aortic dissecting aneurysms and the analysis of stress distribution on vessel wall [23], which are also our future work.

## Figures and Tables

**Table 1:** The image time-domain iteration method.

1. Delineate the region-of-interest
2. Extract the images in the first cardiac cycle after the contrast agent enters the region-of-interest
3. Image denoising with the median filter
4. Extract the time-weighted image, **p**_**i**_: **p**_**i**_=**W**_**j**_ × Δ*t*_*j*_
5. Image time-domain integration, **b**=∑_*j*=0_^*M*−1^**p**_**i**_
6. Extract the TDI image in the region-of-interest

**Table 2:** The mean value, standard deviation, and coefficient of variation of DSA images of the aortic dissection for the patient in Figure 1 in the first cardiac cycle after the contrast agent enters the aortic dissection.

Time	0	1/6*T*	1/3*T*	1/2*T*	2/3*T*	5/6*T*	*T*	TDI image
Mean	0.6794	0.6340	0.6948	0.6507	0.6958	0.5965	0.5978	0.6025
Standard deviation	0.3585	0.3452	0.3473	0.3554	0.3430	0.3784	0.3835	0.4425
Coefficient of variation (%)	52.77	54.44	49.98	54.62	49.30	63.44	64.15	73.44

## Data Availability

The data used to support the findings of this study are available from the corresponding author upon request.
